# Effect of strontium-containing on the properties of Mg-doped wollastonite bioceramic scaffolds

**DOI:** 10.1186/s12938-019-0739-x

**Published:** 2019-12-11

**Authors:** Su Wang, Linlin Liu, Xin Zhou, Danfeng Yang, Zhang’ao Shi, Yongqiang Hao

**Affiliations:** 1 School of Mechanical Engineering, Sichuan University, Chengdu, 610065 Sichuan China; 2 Shanghai Key Laboratory of Orthopaedic Implants, Department of Orthopaedics, Shanghai Ninth People’s Hospital, Shanghai Jiao Tong University School of Medicine, Shanghai, 200011 China

**Keywords:** Direct ink writing 3D printing, Bioceramic scaffolds, Compression strength, Strontium

## Abstract

**Background:**

Bone scaffold is one of the most effective methods to treat bone defect. The ideal scaffold of bone tissue should not only provide space for bone tissue growth, but also have sufficient mechanical strength to support the bone defect area. Moreover, the scaffold should provide a customized size or shape for the patient’s bone defect.

**Methods:**

In this study, strontium-containing Mg-doped wollastonite (Sr-CSM) bioceramic scaffolds with controllable pore size and pore structure were manufactured by direct ink writing 3D printing. Biological properties of Sr-CSM scaffolds were evaluated by apatite formation ability, in vitro proliferation ability of rabbit bone-marrow stem cells (rBMSCs), and alkaline phosphatase (ALP) activity using β-TCP and Mg-doped wollastonite (CSM) scaffolds as control. The compression strength of three scaffold specimens was probed after completely drying them while been submerged in Tris–HCl solution for 0, 2,4 and 6 weeks.

**Results:**

The mechanical test results showed that strontium-containing Mg-doped wollastonite (Sr-CSM) scaffolds had acceptable initial compression strength (56 MPa) and maintained good mechanical stability during degradation in vitro. Biological experiments showed that Sr-CSM scaffolds had a better apatite formation ability. Cell experiments showed that Sr-CSM scaffold had a higher cell proliferation ability compared with β-TCP and CSM scaffold. The higher ALP activity of Sr-CSM scaffold indicates that it can better stimulate osteoblastic differentiation and bone mineralization.

**Conclusions:**

Therefore, Sr-CSM scaffolds not only have acceptable compression strength, but also have higher osteogenesis bioactivity, which can be used in bone tissue engineering scaffolds.

## Background

Reconstruction of bone tissue caused by trauma or tumor, especially large segments of load-bearing bone tissue reconstruction, still faces great challenges for clinicians. In recent years, bone scaffold has become the most effective treatment for bone defects [1]. Compared with cancellous bone reconstruction, cortical bone reconstruction not only requires good bone inductivity of bone scaffolds to stimulate bone formation and angiogenesis, but also has compress strength matching with cortical bone. As a candidate bone implants material, calcium silicate (wollastonite) bioceramics is known for its excellent biocompatibility [2]. Compared with hydroxyapatite and calcium phosphate, wollastonite has higher bioactivity, osteoinductivity and osseointegration [3]. Moreover, Ca and Si ions released by wollastonite have a synergistic effect on the cell proliferation and differentiation of bone [4]. However, the compression strength of wollastonite scaffold fabricated is 20 MPa [5] which is only within the range of cancellous bone (11–24 MPa) [6], therefore it cannot satisfy the repair of bone defects in load-bearing areas.

Many studies have shown that the addition of different bioactive metal ions such as strontium and magnesium into bone scaffolds materials can promote the formation of bone [5, 7–10]. The release of these ions in biomaterials also helps to enhance biological interactions in vitro and in vivo [11, 12]. Magnesium ion and strontium ion play different roles in different stages of bone formation [13]. Strontium, a trace element in human bones, has been shown to stimulate bone formation and reduce bone absorption [12]. In recent years, the use of strontium ions to improve bioceramics has attracted more attention [8]. Xing [14] showed that strontium ions replacing calcium ions in calcium silicate can stimulate the proliferation, osteogenesis and angiogenesis of rabbit bone-marrow stem cells (rBMSCs). Strontium has been increasingly used in bone repair materials to improve the biological activity of bone reconstruction. In addition, the release of strontium, magnesium and silicon in biological materials can improve the biological activity of the materials [15, 16]. Zhang et al. [17] demonstrated that 5 mol% Ca substituted by Sr in calcium silicate had a good biological properties. Magnesium ion is an important element in human body, which is closely related to the proliferation, differentiation and bone mineralization of osteoblasts [18, 19]. Studies have shown that the incorporation of magnesium can change the degradation rate of calcium silicate ceramics, induce the formation of apatite and form a close bond between scaffold and bone tissue [20]. Previous studies have confirmed that biomaterials that form apatite in simulated body fluids (SBF) can bond with living bone through apatite in vivo [21]. The apatite layer contains calcium and phosphate that is necessary for bone growth [22]. Xie et al. [23, 24] used additive manufacturing (3D printing) to manufacture magnesium-doped calcium silicate. The results showed that the obtained bioceramics scaffold had ultrahigh strength when 10 mol% Ca was replaced by Mg [5, 23, 24].

**Fig. 1:**
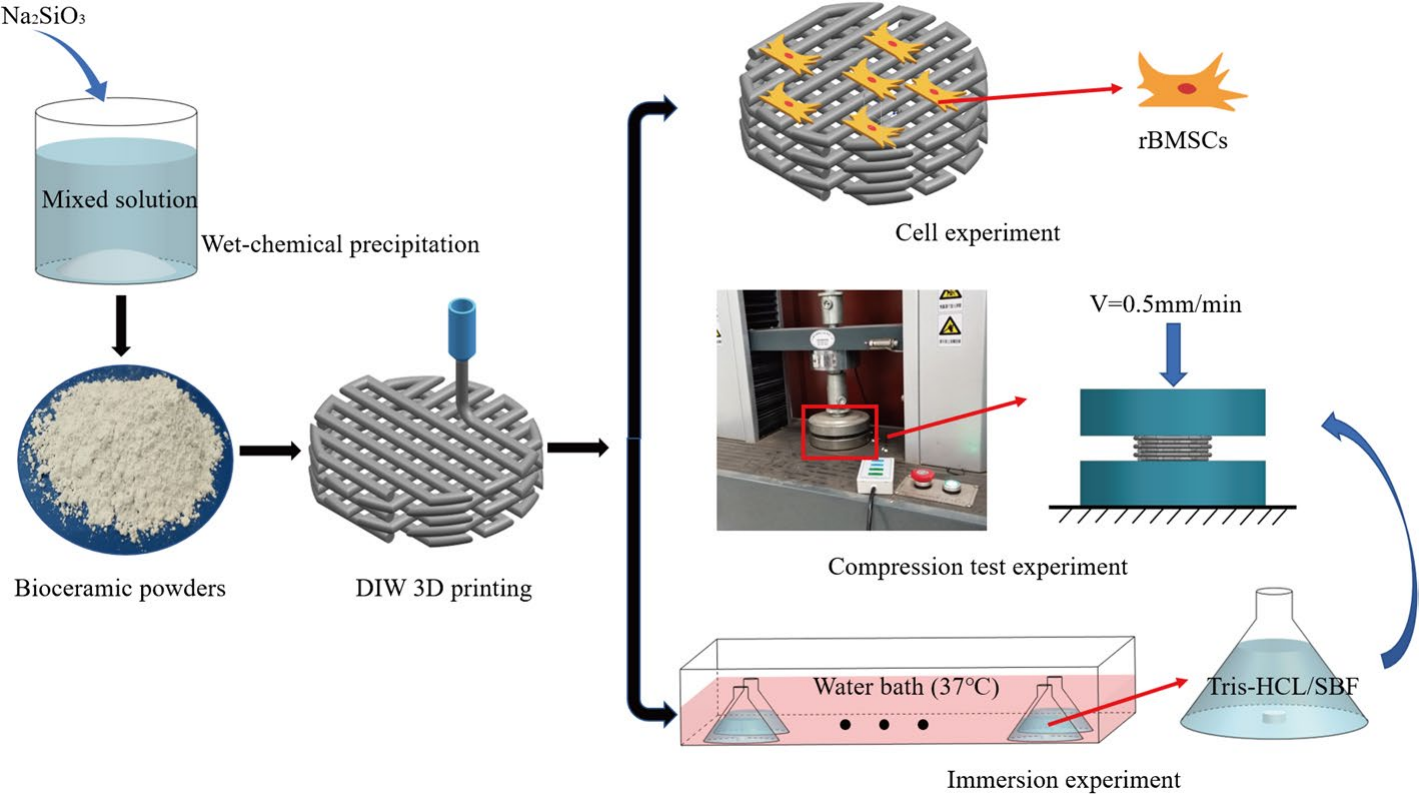
Schematic diagram of experimental setup of bioceramic scaffold

3D printing has been widely used in bone tissue implants, which can be directly linked to CAD models to print the designed geometry, pore size and porosity [6]. Direct ink writing (DIW) is one of the 3D printing technologies, which works by pre-mixing ceramic powder with an organic solvent to form a bio-ink. This is then extruded layer by layer through a printing tube to create a designed porous structure [25]. Personalized medicine needs to develop customized scaffold for patients to match the individualized bone defect shape and tailored biofunction. DIW has excellent performance in bioceramic scaffold printing, and has higher flexibility in controlling scaffold composition and geometric structure [26]. Many studies have personalized repair of rabbit skull, femur and mandibular defects by DIW method [5, 8, 27]. The results showed that the bone scaffolds manufactured by DIW had promising osteogenic and mechanical properties, and had an excellent application in customized bone scaffolds. This study aims to determine the effect of strontium on the rBMSCs proliferation and differentiation on Mg-doped wollastonite (CSM) scaffolds, and to provide a basis for the study of bone tissue engineering scaffolds. Strontium-containing Mg-doped wollastonite (Sr-CSM) were prepared by DIW, and compared with CSM and β-TCP scaffolds in terms of compression strength, in vitro degradation properties and cell proliferation and differentiation properties (Fig. 1).

## Results

### Characterization of bioceramic powders and scaffolds

**Fig. 2:**
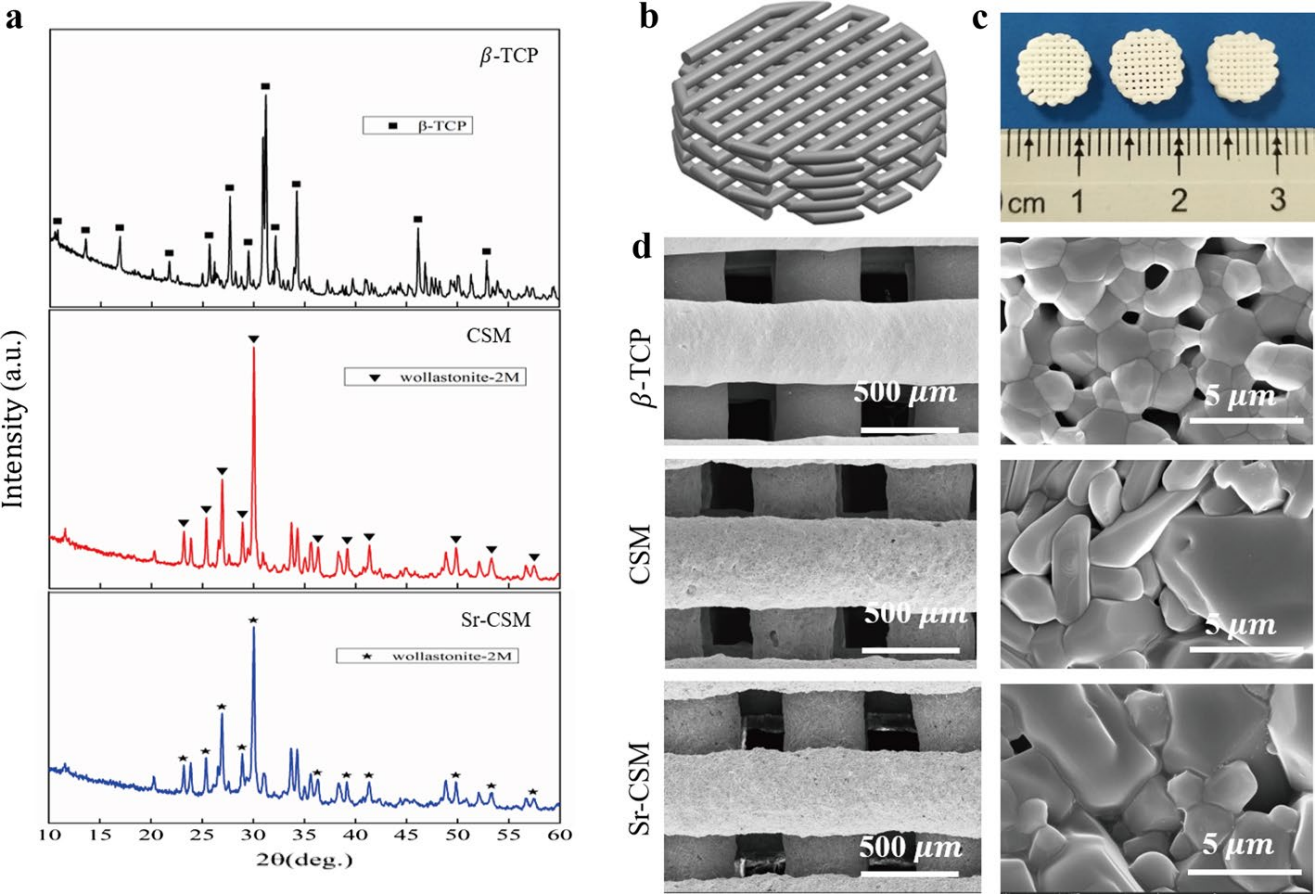
Characterization of the β-TCP, CSM and Sr-CSM powders and scaffolds. **a** XRD patterns of three bioceramic scaffolds, **b** CAD model of three bioceramic scaffolds, **c** digital photograph of three bioceramic scaffolds: from left to right is β-TCP, CSM and Sr-CSM, **d** SEM images of three bioceramic scaffolds

The content of Mg in CSM and Sr-CSM was 2.05 wt% and 2.1 wt%, respectively, which was very close to the theoretical value, while the content of Sr in Sr-CSM was 2.5 wt%, which was slightly lower than the theoretical value. The XRD patterns of the three powders are shown in Fig. 2a. In the figure, it can be seen that the β-TCP powder shows high crystallization of β-TCP (PDF# 09-0189). CSM powder and Sr-CSM powder are mixed with Mg and Sr ions, but the phase of the two powders is still wollastonite-2M (PDF# 27-0088) phase.

The three-dimensional model of three bioceramic scaffold is shown in Fig. 2b. The three types of bioceramic scaffolds (∅8 × 3 mm) sintered after DIW are shown in Fig. 2c. All the three types of scaffolds have a highly controllable macroscopic, and the pore size is about 300 µm (Fig. 2d). As evident in Fig. 1c, the surface of the three types of scaffolds manufactured by DIW is smooth without obvious bubbles. The pore size is smaller than the theoretical value, which may be caused by the shrinkage of the diameter of the strut in the sintering process. High magnification SEM images showed that grain boundaries on the surfaces of the three scaffolds were clearly visible. Compared with CSM scaffolds, the β-TCP grain boundaries showed more gaps, while Sr-CSM scaffolds showed densification grain structure, but did not show grain growth.

### Mechanical properties

**Fig. 3:**
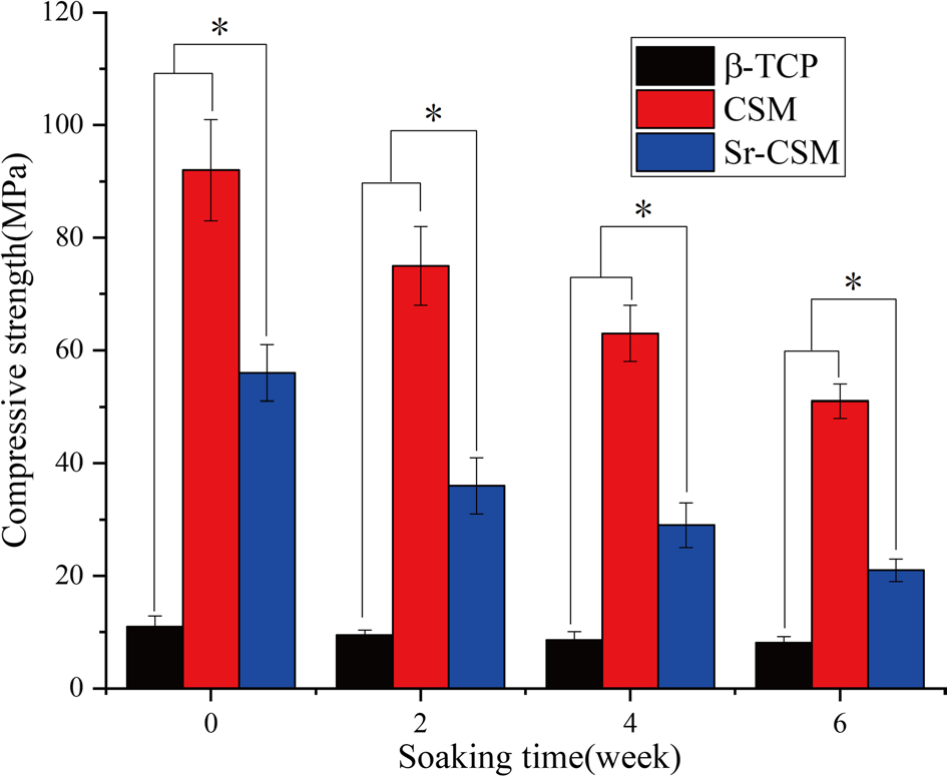
The compression strength of the three scaffolds varies with the soaking time (*p < 0.05)

The compression strength of the three bioceramic scaffold changes with the immersion time is shown in Fig. 3. As can be seen from the figure, although the initial compression strength (56 MPa) of Sr-CSM scaffolds was lower than that of CSM scaffolds (92 MPa), it was far higher than that of traditional β-TCP scaffolds (11 MPa). This could be explained by the similar densification grain structure of Sr-CSM scaffolds and CSM scaffolds (Fig. 2d). As the immersion time increases, the compressive strength of the three scaffolds decreases. Different from the β-TCP scaffolds, the compressive strength of CSM and Sr-CSM scaffolds decreases greatly, while the compression strength of the β-TCP scaffolds remains almost unchanged. However, at week 6, Sr-CSM scaffolds could also maintain a high compressive strength (21 MPa), which was more than two times higher than that of β-TCP scaffolds.

### Biodegradation and apatite-forming ability evaluation

**Fig. 4:**
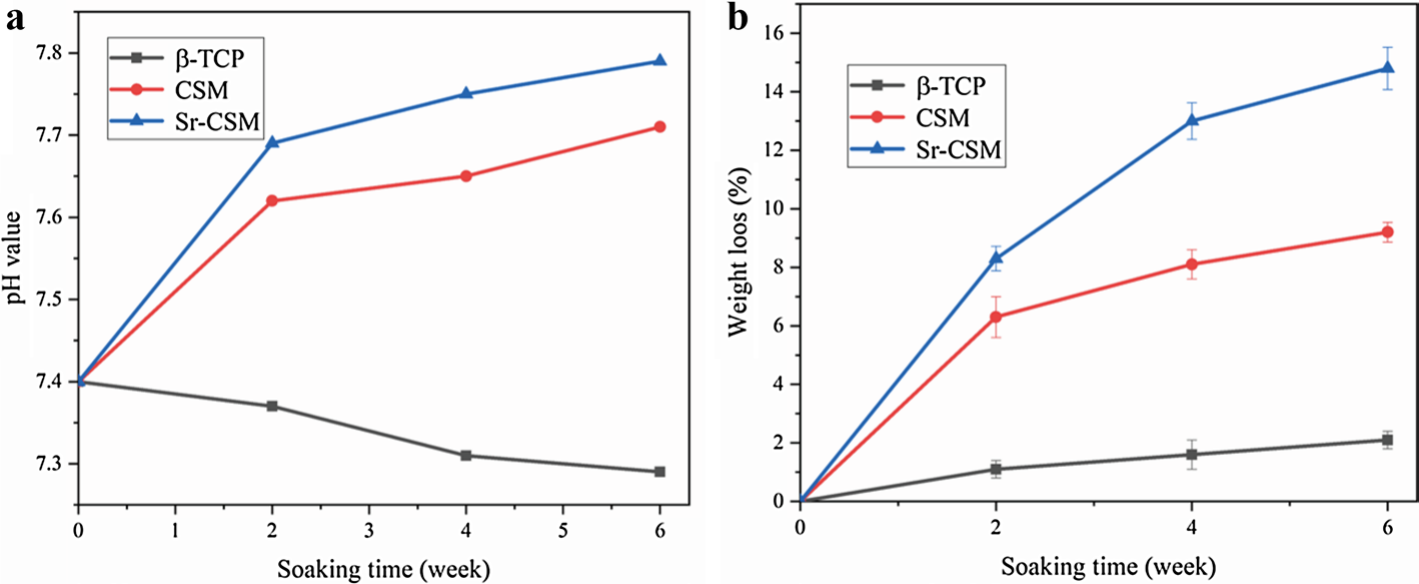
pH value and weight loss of the three scaffolds soaked at each time point. **a** pH value, **b** weight loss

Figure 4 shows the pH values and weight loss of the three scaffolds immersed in Tris–HCl for different times. As visible in Fig. 4a, the pH value of CSM and Sr-CSM scaffolds gradually increased with the increase of soaking time, which was 7.7 and 7.8 after 6 weeks, respectively. For bone tissue growth, the alkaline environment is conducive to the growth and development of bone tissue [28]. The CSM and Sr-CSM scaffolds showed sustained weight loss with increasing immersion time, with a weight loss of 9.2% and 14.8% after 6 weeks, respectively, suggesting that the introduction of Sr accelerated material biodegradation. Meanwhile the weight loss of the β-TCP scaffolds was small (~ 2%).

With the increase of soaking time, the reduction of compression strength was consistent with the weight loss. During 0–4 weeks, the compression strength of CSM and Sr-CSM scaffolds was reduced by 32% and 44%, respectively, and the corresponding weight loss was 8.1% and 13%. During 4–6 weeks, the degradation rate of scaffolds slows down.

**Fig. 5:**
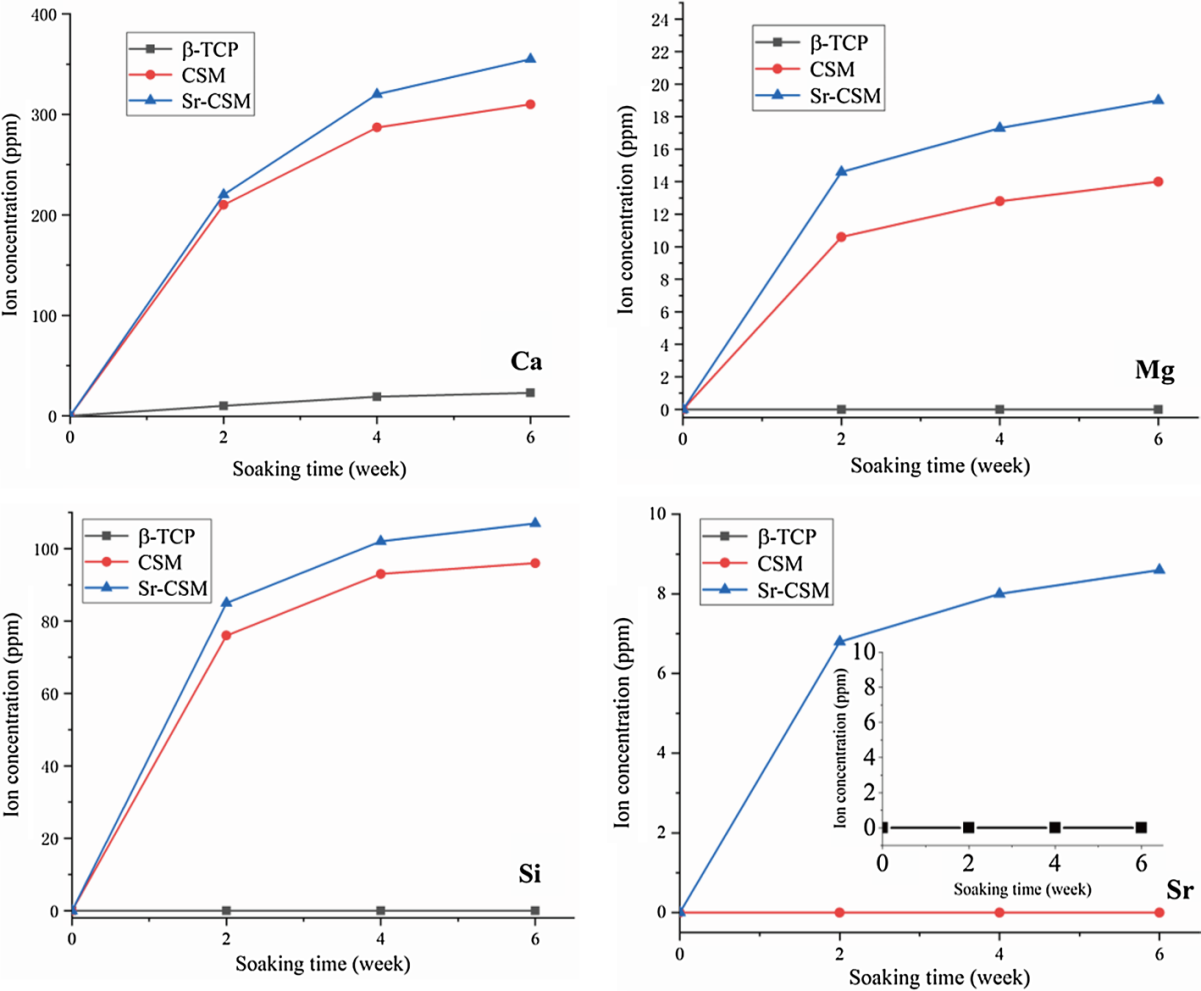
The release of Ca, Mg, Si and Sr ions of three scaffolds soaked in Tris–HCl solution at different time points

Figure 5 shows the release of Ca, Mg, Si and Sr ions of the three scaffolds in Tris–HCl solution at different time points. The concentration of Ca, Mg and Si ions in CSM and Sr-CSM scaffolds gradually increased with the increase of immersion time. Meanwhile, the concentration of Sr ions in Sr-CSM scaffolds also increased continuously. This indicates that the CSM and Sr-CSM scaffolds have sustained ion release during the immersion time. On the contrary, there was only a small increase in ion concentration of the β-TCP scaffolds, which was caused by the slow degradation rate of the β-TCP scaffolds. The release concentrations of Ca, Mg and Si ions of Sr-CSM scaffolds were higher than those of CSM scaffolds at all time points, which was because the degradation rate of Sr-CSM scaffolds was higher than that of CSM scaffolds.

**Fig. 6:**
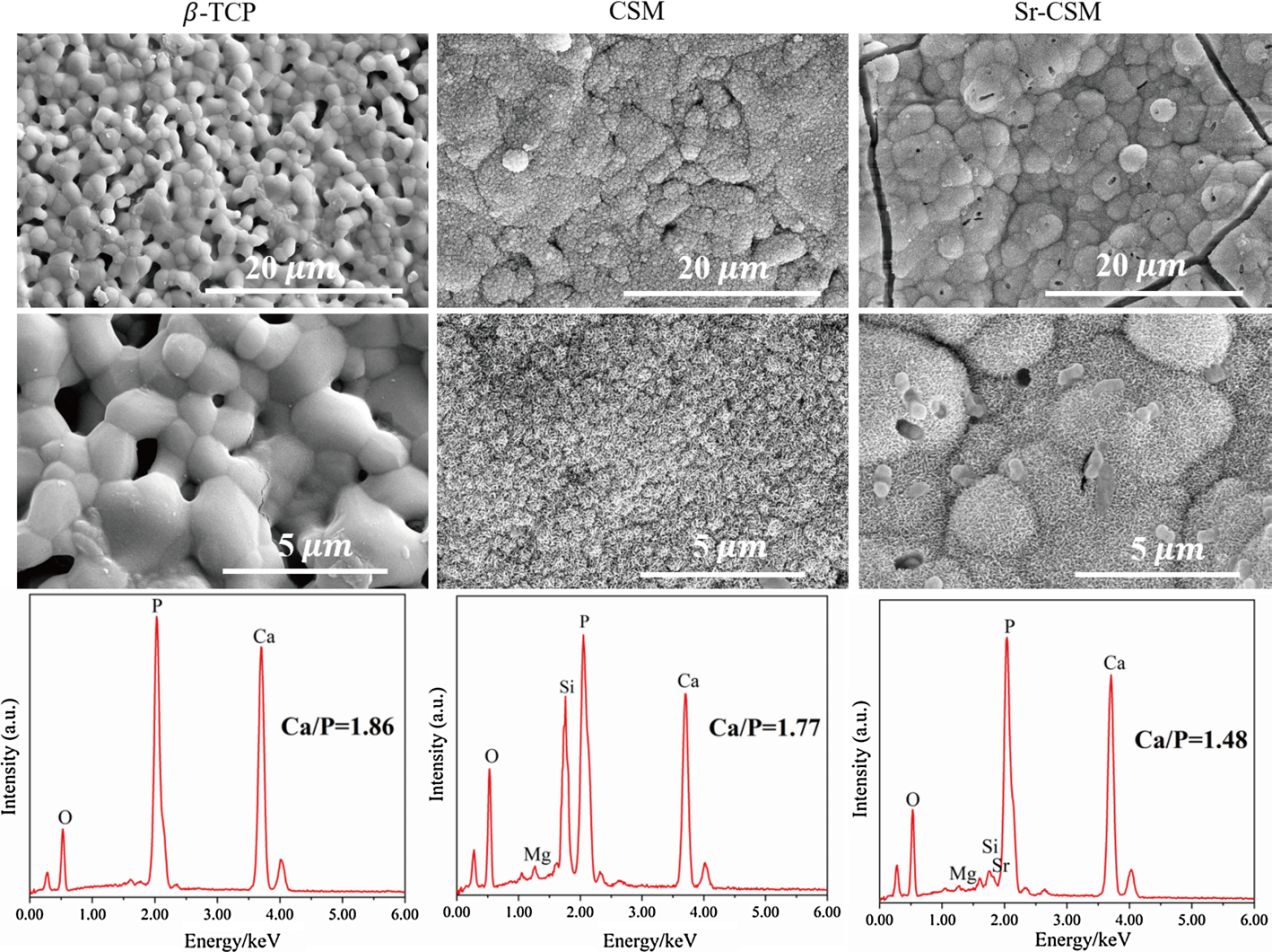
SEM images and EDS analysis results of three scaffolds soaked in SBF for 14 days

In order to determine the surface bioactivity of scaffolds, three scaffolds were immersed in SBF for 14 days. Figure 6 shows SEM images of the three scaffolds soaked in SBF for 14 days. Compared to before immersion (as shown in Fig. 1c), CSM scaffolds and Sr-CSM scaffolds have obvious changes. Both the surface of CSM and Sr-CSM scaffolds had spherical structure precipitation, which was covered by a typical biomimetic apatite, while the spherical particles precipitation on the surface of the β-TCP scaffolds was not obvious. In addition, the surface Ca/P ratio of EDS was 1.48–1.86, and the standard hydroxyapatite Ca/P ratio was 1.67. Studies have shown that the Ca/P ratio between 1.3 and 2.0 can be classified as hydroxyapatite [20], indicating the formation of hydroxyapatite on the surface of Sr-CSM scaffold. The thickest hydroxyapatite layer formed on Sr-CSM scaffolds has been hardening, indicating that Sr-CSM scaffolds have strong apatite mineralization ability.

### The osteogenic bioactivity of rBMSCs in bioceramic scaffolds

**Fig. 7:**
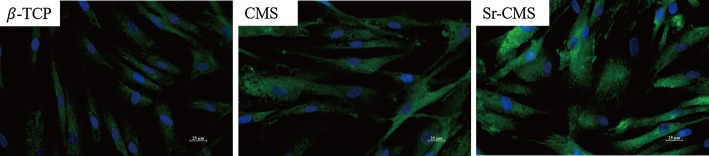
Cell morphologies of the three scaffolds at 7 days of culture: cytoskeleton stained (green) nuclei stained (blue)

The morphology of cells on the three scaffolds is shown in Fig. 7. After 7-day culture, it can be seen that cells are evenly distributed on the three scaffolds. The cells on the CSM and Sr-CSM scaffolds were elongated and had elongated actin filaments (blue). In contrast, the cell density on the β-TCP surface was reduced.

**Fig. 8:**
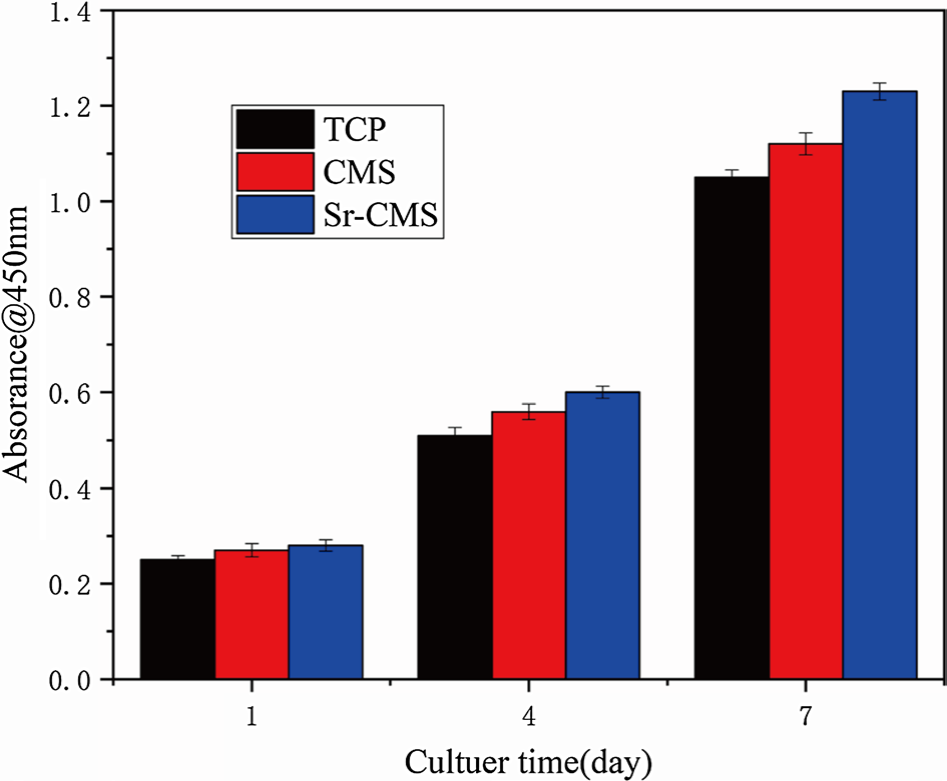
Cell proliferation of the three scaffolds at 1, 4, and 7 days

Cell proliferation on different scaffolds was measured by CCK-8 method at 1, 4, 7 days of culture, as shown in Fig. 8. The cell proliferation on Sr-CSM scaffolds was slightly higher than that on CSM and β-TCP scaffolds on day 1, but not significant. From day 1 to day 4, the number of cells on the Sr-CSM scaffolds was not significantly higher than that of the other two groups. However, the number of cells on Sr-CSM scaffolds was 10% and 18% higher than that on the β-TCP scaffolds and CSM scaffolds, respectively, at day 7, indicating that Sr-CSM scaffolds were more suitable for cell growth than the other two groups of scaffolds.

**Fig. 9:**
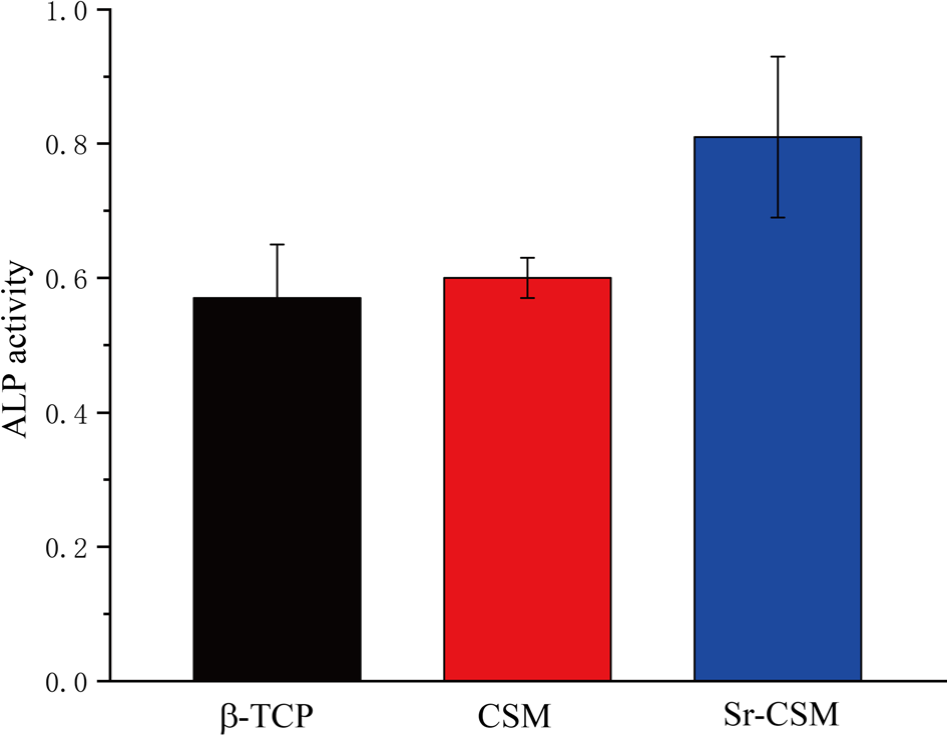
The ALP activity of rBMSCs cultured on β-TCP, CSM and Sr-CSM scaffolds for 14 days

ALP activity is considered to be an early marker of osteoblastic differentiation and bone mineralization [18, 29]. Figure 9 depicts the ALP activity of cells after 14 days on three scaffolds. As can be seen from the figure, ALP activity on Sr-CSM scaffolds is much higher than that on CSM and β-TCP scaffolds, indicating that appropriate Sr ion concentration can stimulate cell differentiation.

## Discussion

Although more and more bioactive materials have been proved to be suitable for bone tissue repair, most of these materials have poor compression strength. For the repair of large segments of load-bearing bone tissue, the bone tissue scaffold should not only provide space for the growth and attachment of cells, but also provide a mechanical support. The force on the scaffold also has a great influence on the stimulation of cell proliferation and differentiation [30]. It is generally believed that the pore size of the scaffold is recommended to be between 150 µm and 400 µm [31, 32], 3D printing technology can create porous scaffolds with controllable pore size and structure. The interconnected pore promotes the circulation of nutrients and oxygen in the pore, making the cells easy to attach to the strut. As the immersion time increases, three kinds of bioceramic scaffolds can maintain their structural integrity, which provides an important place for the growth of bone tissue regeneration in the late stage. The high compression strength and time-varying strength stability of the Sr-CSM scaffold can provide initial structure and stable mechanical support for the generation of bone tissue. The compression strength of bioceramic scaffolds will gradually decrease during 0–6 weeks in vivo, but with the growth of new bone tissue, the compression strength of scaffolds will gradually increase during 6–12 weeks [5]. Therefore, Sr-CSM scaffolds can provide a stability mechanical support for bone tissue growth.

Traditional CSM scaffolds introduce Mg ions into calcium silicate to improve the compression strength of calcium silicate scaffolds, but do not consider to further improve the osteogenic bioactivity of scaffolds. Studies have shown that strontium ions and magnesium ions have synergistic effects on the growth and repair of bone tissue [33]. Therefore, this study enhances the biological performance of CSM scaffolds by introducing strontium.

The production capacity of apatite in SBF is very helpful to predict the osteogenic properties of the material in vivo. Apatite on the surface of the material also helps enhance the ability of bone tissue cells to attachment and differentiation [21], so it is reasonable to believe that Sr-CSM scaffolds are more conducive to binding with host bone tissue when implanted in vivo. The advantages and disadvantages of the three bioceramic scaffolds are shown in Table 1.

**Table 1:** Advantages and disadvantages of three bioceramic scaffolds

Scaffold	Advantages	Disadvantages
β-TCP	Excellent biocompatibility and bone conductivity	Low mechanical strength and cell differentiation ability; poor induction of apatite formation and degradation ability
CSM	Ultrahigh strength; excellent osteogenic properties	Poor induction of apatite formation ability; low capability of cell differentiation
Sr-CSM	Superior ability to induce apatite formation, cell proliferation and differentiation; acceptable mechanical strength	Mechanical strength needs to be improved

In terms of biological properties, some studies have shown that adding a small amount of strontium in scaffolds can greatly improve the osteogenesis bioactivity of scaffolds [17]. In this paper, 2.5% strontium was added into traditional high-strength bioceramic scaffolds. The experimental results showed that the cell activity of Sr-CSM scaffolds was slightly higher than that of the other two groups. The ALP activity was much higher than that of traditional CSM and β-TCP scaffolds (*p* < 0.05). Cell proliferation and differentiation on scaffolds are influenced by many factors, such as ion concentration in microenvironment and pH value. Statistical analysis show that *p* < 0.05 indicated that the difference due to chance is below 5%. The content of strontium in human bones is not very high. Appropriate amount of strontium ions can promote cell proliferation and differentiation, but excessive release of strontium ions in the degradation process of scaffolds will cause harm to human bodies [34]. Sr-CSM scaffolds added strontium ions to CSM by chemical precipitation method, which enabled the scaffolds to have stable compressive strength, continuous and stable strontium ion release and appropriate pH value in vitro degradation experiments. These microenvironments enabled the cells to have better activity compared with other two groups.

In recent years, the application of bioceramic scaffolds in bone tissue repair has become a prime focus of many scholars [35]. These researches focus on improving the biological properties of materials, but there is little research on their mechanical strength. Acceptable mechanical strength of scaffolds is very important for the repair of load-bearing bone tissue. Based on high-strength CSM scaffolds, Sr-CSM scaffolds with controllable structure shape and pore size were successfully prepared by DIW by adding strontium ions into scaffold materials by chemical precipitation method. Through SEM observation, it was found that the addition of a small amount of strontium ions did not change the original material structure itself. Mechanical experiments also confirmed that Sr-CSM scaffolds also had high mechanical strength. After 14 days of SBF immersion, Sr-CSM scaffolds were found to have good apatite formation ability, which indicated that Sr-CSM scaffolds had good binding ability with bone tissue after implantation. The experiment of cell proliferation ability showed that the addition of strontium improved the cell activity of scaffolds, which was slightly higher than the other two groups after 4 days and 7 days, indicating that strontium ions also promoted cell proliferation. ALP activity was used to evaluate the ability of cell differentiation. The results showed that the ALP activity of the Sr-CSM scaffold was significantly higher than that of the other two groups, indicating that the addition of strontium promoted cell differentiation.

In terms of mechanical strength, the introduction of strontium reduced the compressive strength of CSM scaffolds, however it was still five times higher than conventional β-TCP scaffold. In terms of degradation performance, the introduction of strontium accelerated the degradation rate of Sr-CSM scaffolds. This was accompanied by a large weight loss at each time point and a decline in mechanical properties. However, it can still maintain a relatively stable mechanical strength at each time point. For in situ load-bearing bone repair (e.g., osteonecrosis of the femoral head), high strength is favorable for better insert, however more important is the rapid induction of osteogenesis. Sr-CSM scaffold had sufficient initial strength, and the compression strength of the scaffold after 6 weeks of immersion in Tris–HCl was still twice than that of the conventional β-TCP scaffold. Therefore, it is reasonable to believe that Sr-CSM scaffold can provide good mechanical support for bone tissue in in situ load-bearing bone repair. On this basis, Sr-CSM scaffold has significantly better apatite formation ability and bone induction performance than the β-TCP scaffold and CSM scaffold, and can quickly induce osseointegration. In conclusion, the acceptable compression strength and osteogenesis bioactivity make Sr-CSM scaffolds as a potential candidate for hard tissue implants.

## Conclusions

In this study, strontium was added into CSM bioceramic scaffolds to obtain a bioceramic scaffolds with satisfactory strength and biological properties. The scaffolds prepared by DIW have interconnected porous structures, and the shape and size of the pore can be controlled. After 6 weeks of Tris–HCl immersion, Sr-CSM scaffolds showed sustained ion release ability. In addition, Sr-CSM scaffolds showed good apatite formation ability and ability to induce cell proliferation and differentiation in vitro due to the introduction of strontium. Therefore, Sr-CSM scaffolds are expected to be candidates for the repair of large segments bone tissue.

## Materials and methods

### Preparation of powders

CSM and Sr-CSM powders were synthesized by a wet-chemical precipitation method. CSM powder replaces 10 mol% of Ca in wollastonite with Mg, and Sr-CMS replaces 15 mol% of Ca in wollastonite with 10 mol% Mg and 5 mol% Sr. Briefly, Ca(NO_3_)_2_ and Na_2_SiO_3_ dissolved in de-ionized water in the concentration of 0.5 mol/l with certain proportion of Ca(NO_3_)_2_ replaced by Mg(NO_3_)_2_ and Sr(NO_3_)_2_. Then the Na_2_SiO_3_ solution was dropped into the Ca (NO_3_)_2_/Mg (NO_3_)_2_/Sr (NO_3_)_2_ solution mixture under continuous stirring. Washed with de-ionized water three times to remove impurity ions, reoccupy anhydrous ethanol washing three times. Dry powder at 80 °C for 24 h, and then under the 950 °C calcination 120 min. The as-calcined bioceramic powder is ground in a planetary ball mill for 4 h. The particle size of the resulting bioceramic powders was below 10 µm. The β-TCP powder is bought from Xi’an Particle Cloud Biotechnology Co., Ltd., and the particle size was also below 10 µm.

### DIW 3D printing preparation of scaffolds

The bioceramic ink for layer-by-layer printing of β-TCP, Sr-CSM and CSM scaffolds(∅8 × 3 mm) was prepared by mixing 14.0 g of powder with 5.0 g of 6% polyvinyl alcohol (PVA, Sigma-Aldrich) solution. After stirring, add the bio-ink to the printing tube, and bioceramic scaffolds with 0^°^/90^°^ strut pore structure were prepared by EFL-BP6800 (Suzhou Intelligent Manufacturing Research Institute, SuZhou, China). The ink is extruded by moving the piston rod, and the nozzle moves at a speed of 6 mm/s. The nozzle diameter and filament spacing were 450 µm and 850 µm, respectively. Dry as-built scaffolds at 80 °C for 24 h and sintered samples by two-step sintering method [36] (the heating rate is 3 °C/min and held at 1150 °C for 45 min, then cooled down to 1060 °C within 10 min and held for 3 h).

### Characterization of powders and scaffolds

The phase composition of the bioceramic powders was verified by X-ray diffraction (XRD, Rigaku Co., Japan) at 40 kV/40 mA. Data were collected between 10 °C and 80 ℃ on a 2θ scale in step of 0.02 °C and 1.5 s per step. Inductively coupled plasma-optical emission spectrometry (ICP-OES; Thermo) was used to measure the chemical composition of the bioceramic powders.

The morphology of scaffolds was observed by scanning electron microscopy (SEM, JSE-5900LV, Japan). The apatite mineralization ability of scaffolds was investigated by SBF [21]. The bioceramic scaffolds (*n* = 4,∅8 × 3 mm) were immersed in SBF with a SBF solution volume to scaffold mass ratio of was 200 mL/g at 37 °C for 14 days, the SBF solution kept unaltered during immersion. After soaking, samples were washed three times with ethanol and was observed by SEM.

### Mechanical property of scaffolds

The compressive strength of scaffolds (*n* = 4,∅8 × 3 mm) were measured by an Electro-mechanical Universal Testing Machine (CTM8180, Xie Qiang Instrument Manufacturing), and samples were tested with a constant displacement rate of 0.5 mm/min. The compression strength of three scaffolds at 0, 2, 4, 6 weeks in Tris–HCl was measured after complete drying.

### Biodegradation property of scaffolds

The β-TCP, Sr-CSM and CSM scaffolds (*n* = 4,∅8 × 3 mm) were immersed in Tris–HCl solution (pH 7.4) with a solution volume to scaffold mass ratio of 200 mL/g at 37 °C. After 2, 4, 6 weeks of soaking, each sample was completely dried and weighed. The weight loss (WL) was expressed as:

$${\text{WL}}\% = (\omega_{0} - \omega_{t} )/\omega_{0} \times 100\% ,$$

where the $\omega_{0}$ is the initial weight of the sample, $\omega_{t}$ is the sample weight at week *t*. Ion concentration and pH value of solution at each time were measured by pH-meter and ICP-OES, respectively.

### The osteogenic activity of rBMSCs in bioceramic scaffolds

#### Cell culture

The rBMSCs was obtained from JRDUN Biotechnology (Shanghai) Co., Ltd, and applied in the following experiments. rBMSCs were cultured in DMEM with 10% fetal bovine serum and 1% penicillin/streptomycin at 37 °C under 5% CO_2_ humidified atmosphere.

#### Cell proliferation and attachment

The proliferation of rBMSCs in response to CSM, Sr-CSM and β-TCP scaffolds were determined using a cell counting Kit-8 (CCK-8, Beyotime). rBMSCs (10^4^ cells/scaffold) seeded on each scaffold. Culture medium is changed every 2 days during the experiment. After culturing for 1, 4 and 7 days, cells were harvested for CCK-8 assay according to the manufacturer’s guidelines (three samples for each group). The absorbance of this solution was quantified by spectrophotometer at 450 nm with a plate reader (Bio TEK Instrument, EL307C).

To intuitively observe the growth of cells on the scaffold, the cell morphology of three kinds of scaffold was observed via laser scanning confocal microscopy (LSCM). Briefly, the cell slides were washed with PBS to remove the medium, fixed with 4% formaldehyde for 30 min, and washed with PBS for three times. The cells were permeabilized with 0.5% Triton X-100 (Solarbio) for 10 min and washed with PBS for three times. The cells were blocked with 1% bovine serum albumin (Solarbio) for 1 h and washed with PBS for 3 times. Cytoskeleton and nucleus were labeled with FITC-conjugated phalloidin and DAPI, respectively, for 40 min in PBS solution. Then LSCM was used to photograph the cells.

#### Alkaline phosphatase (ALP) activity

To investigate the influence of Sr-CSM scaffold on the osteogenic differentiation of cell, rBMSCs (10^5^ cells/scaffold) seeded on each scaffold and culture in osteogenic induction medium for 14 days. The rBMSCs were lysed with 200 μl of 0.2% Triton X-100 and then centrifuged at 14,000 rpm for 5 min at 4 °C. 50 μl of supernatant was mixed with 150 μl working solution, and enzyme activity was determined by ALP kit (Thermo). The absorbance of this solution was quantified by microplate reader at 405 nm with a plate reader. The total protein content was determined via the bicinchoninic acid protein assay kit (Thermo). The relative ALP activity was obtained by the changed OD values divided by the reaction time and the total protein content.

### Statistical analysis

Analysis was performed using SPSS software (SPSS Inc., Chicago, Il, USA). All the data were expressed as mean ± standard deviation and analyzed with the one-way ANOVA. In all cases the results were considered statistically significant with a *p* value less than 0.05.

## Data Availability

The datasets used and/or analyzed during the current study are available from the corresponding author on reasonable request.

## Abbreviations

rBMSCs: rabbit bone-marrow stem cells
CSM: Mg-doped wollastonite
Sr-CSM: strontium-containing Mg-doped wollastonite
PVA: polyvinyl alcohol
XRD: X-ray diffraction
ICP-OES: inductively coupled plasma-optical emission spectrometry
SEM: scanning electron microscopy
ALP: alkaline phosphatase
SBF: simulated body fluids
LSCM: laser scanning confocal microscopy
DIW: direct ink writing
